# Predicting cognitive performance in patients with atrial fibrillation by 24-hour Holter electrocardiography: the potential role of an arrhythmic fingerprint

**DOI:** 10.1093/ehjopen/oeag137

**Published:** 2026-09-08

**Authors:** Andrea Saglietto, Luca Congiu, Rachele Marcato, Martina Amanzio, Cristiano Manco, Eleonora Bertello, Carola Griffith Brookles, Elena Cavallone, Gaia Miceli, Luca Ridolfi, Carla Giustetto, Veronica Dusi, Federico Ferraris, Gaetano Maria De Ferrari, Stefania Scarsoglio, Matteo Anselmino

**Affiliations:** Division of Cardiology, Cardiovascular and Thoracic Department, ‘Città della Salute e della Scienza’ Hospital, Corso Bramante 88, 10126 Torino, Italy; Department of Mechanical and Aerospace Engineering, Politecnico di Torino, Corso Duca degli Abruzzi 24, 10129 Torino, Italy; Department of Medical Sciences, University of Turin, Corso A.M. Dogliotti 14, 10126 Torino, Italy; Department of Psychology, University of Turin, Via Giuseppe Verdi 10, 10124 Torino, Italy; Department of Psychology, University of Turin, Via Giuseppe Verdi 10, 10124 Torino, Italy; Department of Medical Sciences, University of Turin, Corso A.M. Dogliotti 14, 10126 Torino, Italy; Department of Medical Sciences, University of Turin, Corso A.M. Dogliotti 14, 10126 Torino, Italy; Department of Medical Sciences, University of Turin, Corso A.M. Dogliotti 14, 10126 Torino, Italy; Department of Medical Sciences, University of Turin, Corso A.M. Dogliotti 14, 10126 Torino, Italy; Department of Environmental, Land and Infrastructure Engineering, Politecnico di Torino, Corso Duca degli Abruzzi 24, 10129 Torino, Italy; Division of Cardiology, Cardiovascular and Thoracic Department, ‘Città della Salute e della Scienza’ Hospital, Corso Bramante 88, 10126 Torino, Italy; Department of Medical Sciences, University of Turin, Corso A.M. Dogliotti 14, 10126 Torino, Italy; Division of Cardiology, Cardiovascular and Thoracic Department, ‘Città della Salute e della Scienza’ Hospital, Corso Bramante 88, 10126 Torino, Italy; Department of Medical Sciences, University of Turin, Corso A.M. Dogliotti 14, 10126 Torino, Italy; Division of Cardiology, Cardiovascular and Thoracic Department, ‘Città della Salute e della Scienza’ Hospital, Corso Bramante 88, 10126 Torino, Italy; Division of Cardiology, Cardiovascular and Thoracic Department, ‘Città della Salute e della Scienza’ Hospital, Corso Bramante 88, 10126 Torino, Italy; Department of Medical Sciences, University of Turin, Corso A.M. Dogliotti 14, 10126 Torino, Italy; Department of Mechanical and Aerospace Engineering, Politecnico di Torino, Corso Duca degli Abruzzi 24, 10129 Torino, Italy; Division of Cardiology, Cardiovascular and Thoracic Department, ‘Città della Salute e della Scienza’ Hospital, Corso Bramante 88, 10126 Torino, Italy; Department of Medical Sciences, University of Turin, Corso A.M. Dogliotti 14, 10126 Torino, Italy

## Introduction

AF is the most common cardiac arrhythmia in the elderly population and is increasingly recognized as an independent contributor to cognitive decline^1–4^. Several studies have shown that AF patients experience a faster decline in cognitive performance and reach the threshold for dementia earlier than age-matched controls, even in the absence of clinically overt cerebrovascular events.^5^ Cognitive impairment in AF often presents subclinically, mainly involving executive function, attention, working memory, and processing speed, with important consequences on functional autonomy and quality of life.

Among the mechanisms proposed to explain this association, alteration of cerebral haemodynamics related to interbeat interval (RR interval) irregularity has gained increasing attention. In AF, the irregular ventricular response produces beat-to-beat fluctuations in cerebral blood flow, exposing the brain to repeated episodes of transient hypoperfusion and hypertensive peaks^6–8^. Moreover, the irregular pulsatility of arterial cerebral vessels may contribute to impaired brain glymphatic function.^9^

The RR interval dynamics at 24 h of Holter electrocardiography (ECG) recordings might therefore help identify an arrhythmic fingerprint, defined as a characteristic pattern of RR interval variability, associated with cognitive decline in patients with AF, enabling early recognition of individuals at higher risk of cognitive impairment.

The aim of the present pilot study was to evaluate whether a model integrating 24 h of Holter ECG RR pattern on top of clinical variables could improve cognitive function prediction in AF patients, as evaluated by a multidimensional neuropsychological assessment.

## Methods and results

**Figure 1:**
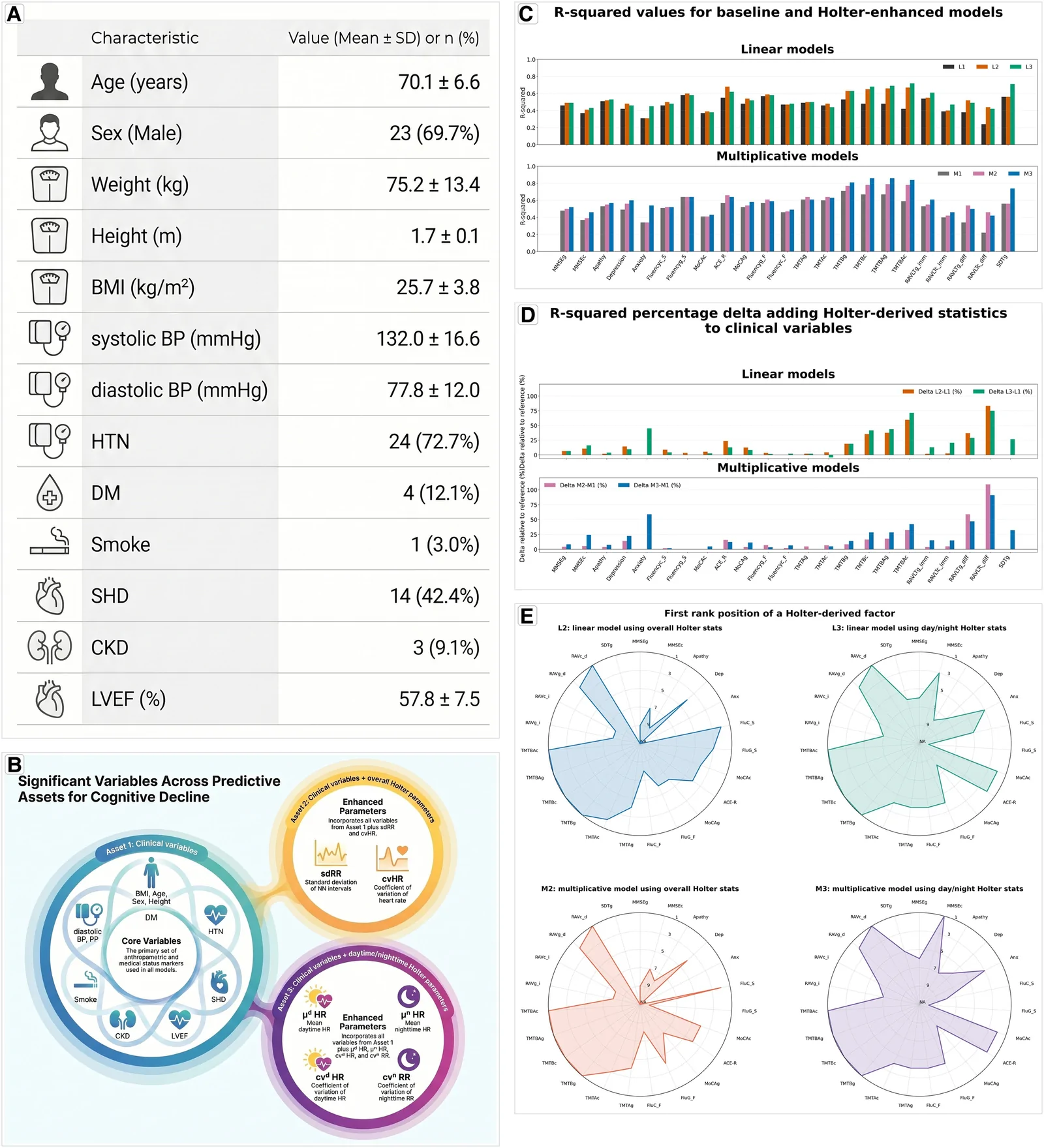
(*A*) Main clinical characteristics of the study population. (*B*) Different regression settings and variables retained as significant after the variance inflation factor-based selection procedure. (*C*) Absolute R^2^ values for baseline and Holter-enhanced models, separately for linear and multiplicative models. (*D*) Corresponding percentage change in ‘R^2^’ after adding Holter-derived statistics to the clinical models. (*E*) Radar plots, for each outcome and model configuration, displaying the first rank position at which a Holter-derived variable appears among the selected regressors. BMI, body mass index; BP, blood pressure; CKD, chronic kidney disease; DM, diabetes mellitus; HR, heart rate; HTN, hypertension; LVEF, left ventricular ejection fraction; SHD, structural heart disease; ACE, Addenbrooke’s Cognitive Examination; Fluency_F, fonemic fluency; Fluency_S, semantic fluency; MMSE, Mini-Mental State Examination; MoCA, Montreal Cognitive Assessment; RAVLT_imm, Rey Auditory Verbal Learning Test—immediate learning; RAVLT_diff, Rey Auditory Verbal Learning Test—delayed recall; STD, Symbol Digit Test; TMT, Trail Making Test.

We prospectively enrolled consecutive patients with ongoing persistent AF undergoing 24 h of Holter ECG monitoring in our centre. Patients with previous stroke, known dementia, severe neurological disorders, or unwillingness to complete neuropsychological testing were excluded. Demographic and clinical variables, including age, sex, body mass index, blood pressure, cardiovascular risk factors, comorbidities, and left ventricular ejection fraction, were collected. The study population consisted of 33 patients with a mean age of 70.1 ± 6.6 years, 70% male (*Figure 1A*).

Mean, standard deviation, and coefficient of variation of RR intervals and heart rate (HR) were calculated over the entire recording, and separately during daytime (4 p.m. to 6 p.m.) and night-time (1 a.m. to 3 a.m.) intervals, selected as representative wakefulness and sleep periods.

All patients underwent comprehensive neuropsychological evaluation according to the National Institute for Neurological Disorders and Stroke–Canadian Stroke Network recommendations. Evaluators were blinded to Holter-derived parameters. Global cognition was assessed using Mini-Mental State Examination (MMSE), Addenbrooke’s Cognitive Examination–Revised (ACE-R), and Montreal Cognitive Assessment (MoCA). Specific cognitive domains were explored using standardized tests investigating executive function, processing speed, attention, language, and memory, including Trail Making Test (TMT-A, TMT-B, and TMT B–A), Symbol Digit Test, phonemic and semantic verbal fluency, and Rey Auditory Verbal Learning Test (RAVLT) (immediate and delayed recall). Mood-related symptoms were also assessed using the Beck Depression Inventory, Apathy Evaluation Scale, and Hamilton Anxiety Rating Scale. Both raw (g) and corrected scores adjusted for age, sex, and education (c) were included in the analysis.

Three regression settings were tested: (1) clinical and demographic variables only; (2) clinical variables plus Holter-derived overall RR/HR parameters; and (3) clinical variables plus separate daytime and night-time Holter parameters. To reduce multicollinearity, regressors were selected using an iterative variance inflation factor (VIF)-based procedure, removing variables with VIF > 5 until all remaining predictors showed acceptable collinearity. In Setting 3, both daytime and night-time Holter parameters were retained, suggesting independent contributions. Multivariate linear and multiplicative regression analyses were performed using the retained predictors to evaluate linear and non-linear associations with neuropsychological performance. Model accuracy was assessed using coefficients of determination (R^2^).

Variables retained after VIF-based selection for the three regression settings are summarized in *Figure 1B*. Clinical variables included age, BMI, blood pressure, and left ventricular ejection fraction, while Holter-derived RR/HR variability parameters were retained in both Settings 2 and 3, confirming their independent contribution beyond clinical characteristics.

As shown in *Figure 1C*, the inclusion of Holter-derived parameters significantly improved predictive performance of the models across most neuropsychological outcomes. Global cognitive tests such as MMSE, ACE-R, and MoCA showed coefficients of determination up to R^2^ ≈ 0.6, with age and RR variability emerging as the strongest predictors. The highest model performance was observed for executive function, particularly for TMT-B and TMT B–A, where multiplicative models reached R^2^ values up to approximately 0.8. In general, multiplicative models outperformed linear models, especially when daytime and night-time Holter parameters were analysed separately (Setting 3).

*Figure 1D* further quantified the incremental value of Holter analysis by showing the percentage increase in R^2^ compared with models based on clinical variables alone. The addition of Holter-derived RR statistics produced the greatest improvement for executive function and memory-related tests, particularly TMT-B, TMT B–A, and RAVLT, indicating that RR interval dynamics provides relevant information not captured by standard clinical assessment alone. In fact, as shown in *Figure 1E*, Holter-derived factors frequently ranked as the first predictive variable in both linear and multiplicative models, supporting that RR dynamics represents a clinically meaningful fingerprint associated with early cognitive impairment in AF patients.

## Limitations

The main limitation of the present study is the relatively small sample size. However, the consistency of the signal across different cognitive domains and regression models supports the biological plausibility of the observed association. In addition, the lack of detailed AF phenotype characterization and treatment-related variables may influence both RR dynamics and cognition. Finally, the exploratory nature of the analysis and the number of cognitive outcomes represent additional limitations.

## Discussion and conclusions

The main finding of this study is that simple Holter-derived parameters significantly improve the prediction of cognitive performance in patients with ongoing AF beyond standard clinical variables alone, with the strongest effect observed for executive function, a domain increasingly recognized as particularly sensitive in ageing and cognitive vulnerability. This is consistent with evidence showing that executive control represents a particularly sensitive domain across ageing trajectories, from healthy ageing to cognitive impairment.^10^

In this context, 24 h of Holter ECG may provide a simple tool to identify arrhythmic patterns associated with cognitive vulnerability in AF patients. Whether such information may contribute to personalized therapeutic strategies, including rhythm-control approaches, requires validation in longitudinal studies.

## Data Availability

The data underlying this article will be shared upon reasonable request to the corresponding author.
